# The Degree of Leukocytosis and Urine GATA-3 mRNA Levels Are Risk Factors for Severe Acute Kidney Injury in Puumala Virus Nephropathia Epidemica

**DOI:** 10.1371/journal.pone.0035402

**Published:** 2012-04-16

**Authors:** Daniel H. Libraty, Satu Mäkelä, Jennifer Vlk, Mikko Hurme, Antti Vaheri, Francis A. Ennis, Jukka Mustonen

**Affiliations:** 1 Department of Medicine, Medical School, University of Massachusetts, Worcester, Massachusetts, United States of America; 2 Department of Medicine, School of Medicine, University of Tampere, Tampere, Finland; 3 Department of Microbiology, School of Medicine, University of Tampere, Tampere, Finland; 4 Department of Virology, Haartman Institute, University of Helsinki, Helsinki, Finland; 5 Center for Infectious Disease and Vaccine Research, Medical School, University of Massachusetts, Worcester, Massachusetts, United States of America; University of Sao Paulo Medical School, Brazil

## Abstract

Puumala hantavirus (PUUV) infection, also known as nephropathia epidemica, is the most common cause of hemorrhagic fever with renal syndrome (HFRS) in Europe. The pathogenesis of PUUV nephropathia epidemica is complex and multifactorial, and the risk factors for severe acute kidney injury (AKI) during acute PUUV infection are not well defined. We conducted a prospective study of hospitalized patients with PUUV infection in Tampere, Finland to identify acute illness risk factors for HFRS severity. Serial daily blood and urine samples were collected throughout acute illness and at 2 week and 6 month convalescent visits. By univariate analyses, the maximum white blood cell count during acute illness was a risk factor for severe AKI. There were no significant associations between PUUV-induced AKI severity and platelet counts, C-reactive protein, or alanine aminotransferase levels. Maximum plasma interleukin (IL)-6, urine IL-6, and urine IL-8 concentrations were positively associated with PUUV-induced AKI. Finally, the maximum urinary sediment GATA-3 mRNA level was positively correlated with the peak fold-change in serum creatinine, regardless of AKI severity classification. By multivariate analyses, we found that the maximum levels of leukocytes and urinary sediment GATA-3 mRNA during acute illness were independent risk factors for severe PUUV-induced AKI. We have identified novel acute illness risk factors for severe PUUV-induced AKI.

## Introduction

Puumala hantavirus (PUUV) infection, also known as nephropathia epidemica (NE), is the most common cause of hemorrhagic fever with renal syndrome (HFRS) in Europe [1]. In Finland, the average annual incidence is 31/100,000 population and the incidence is increasing [2]. The course of the disease may be divided into febrile, hypotensive, oliguric, diuretic, and convalescent phases, but these phases may overlap and the clinical severity varies. Renal involvement results in the need for transient hemodialysis treatment in 5% of hospital-treated NE patients [1], and the case fatality rate is 0.08% [2].

The pathogenesis of PUUV NE is complex and multifactorial with host genetic properties having an impact on disease severity. Human leukocyte alloantigen (HLA) B8 and DRB1*0301 alleles are associated with severe clinical disease, and genetic polymorphisms of the cytokines tumor necrosis factor alpha, interleukin-1 (IL-1), and interleukin-1 receptor antagonist (IL-1Ra) also influence the outcome of infection [3], [4]. The surface density of platelet β_3_ integrin, the cellular receptor of hantaviruses, is associated with disease severity in patients infected with Hantaan virus [5], another HFRS inducing hantavirus. However, the risk factors for severe acute kidney injury (AKI) during acute PUUV HFRS are less well defined. Two reports have found that serum and urine levels of interleukin-6 (IL-6) correlated with PUUV HFRS severity [6], [7]. Another report has shown that high serum indoleamine 2,3-dioxygenase activity was associated with severe AKI in PUUV NE [8]. We conducted a prospective study of hospitalized patients with PUUV infection in Tampere, Finland to identify acute illness risk factors for HFRS severity. Serial daily blood and urine samples were collected throughout acute illness and at 2 week and 6 month convalescent visits. We found that the maximum levels of leukocytes and urinary sediment GATA-3 mRNA [9] during acute illness were independent risk factors for severe PUUV-induced AKI.

## Methods

### Ethics Statement

The Institutional Review Boards of Tampere University Hospital and the University of Massachusetts Medical School approved the study. The study was conducted according to the principles expressed in the Declaration of Helsinki. Patients were recruited and enrolled after providing written informed consent.

### Clinical Study

The study was carried out at Tampere University Hospital, University of Tampere School of Medicine, Tampere, Finland. All patients came from the Pirkanmaa region of Finland and were hospitalized at Tampere University Hospital due to serologically confirmed acute PUUV infection between 2005–2007. Acute PUUV infection was diagnosed on presentation by a positive rapid anti-PUUV IgM test (POC® PUUMALA IgM, Reagena Ltd, Toivala, Finland) and later confirmed by an anti-PUUV IgM EIA (Reagena Ltd, Toivala, Finland). Severe AKI during PUUV infection was defined by a >3-fold rise in serum creatinine (Cr) during acute illness compared to a 6 month baseline value (Acute Kidney Injury Network (AKIN) stage 3 [10]).

Clinical variables and laboratory data were obtained daily throughout hospitalization and were abstracted onto a standardized case report form. The clinical variables included the length of hospital stay (days), length of fever (days), history of smoking, need for transient hemodialysis treatment (yes/no), systolic and diastolic blood pressures (mmHg), heart rate, respiratory rate, daily urinary output (ml), and daily weight (kg). Daily complete blood counts (CBC), plasma CRP levels, serum liver function tests, and serum Cr concentrations were determined at the Laboratory Centre of the Pirkanmaa Hospital District using standard methods. Daily urine Cr and albumin concentrations were determined at the University of Massachusetts Memorial HealthCare Hospital Labs using standard methods. Serial daily morning blood (plasma) and urine were collected and cryopreserved for 5 consecutive days or until the end of hospitalization, whichever came first. Blood and urine samples were also collected at 15±5 days and 180±14 days after hospitalization. The urine was spun at 500 *g*×5 minutes, and cell free urine was cryopreserved. Urinary cell sediment was placed immediately in RNALater™ and cryopreserved.

### Luminex™ analysis

Serial daily plasma and urine analyte concentrations were determined on a Bio-Plex Luminex-100 station at the Baylor NIAID Luminex Core Facility (Dallas, TX) by standard methods. Eight cytokines and chemokines were selected as analytes to examine in plasma and urine over the course of acute PUUV infection: IL-1α, IL-1β, IL-2, IL-6, IL-8, IL-15, CXCL10, and MCP-1. R&D Systems (Minneapolis, MN) Luminex™ reagents were used. An eight point standard curve was run for each analyte, and all analyte concentrations fell on the standard curves.

### Quantitative (q)RT-PCR

Total cellular RNA from urinary sediment was isolated using the RNeasy Kit (Qiagen, Valencia, CA). Urine cellular RNA was pre-amplified using the Taqman® PreAmp Master Mix and probes, per the manufacturer's instructions (Applied Biosystems, Foster City, CA). The selected genes were amplified with similar efficiency. qRT-PCR for a T-cell associated gene, CD3 epsilon (CD3ε), a type 1 cytokine transcription factor (T-bet), a type 2 cytokine transcription factor (GATA-3), and the housekeeping gene β-actin was performed on an ABI 7300 real-time PCR machine, using Taqman® reagents and a standard protocol (Applied Biosystems, Foster City, CA). Each mRNA transcript in each sample was assayed in triplicate. The relative expression of CD3ε, T-bet, and GATA-3 mRNA levels was normalized to the β-actin mRNA level in each sample. Fold-changes in normalized CD3ε, T-bet, and GATA-3 mRNA levels were expressed relative to 2 week convalescent values in each patient using the 2^−ΔΔCt^ method.

### Statistical Analysis

The PASW software package (version 18.0) was used for statistical analyses. For normally distributed variables, comparisons between 2 groups were analyzed using Student's t test. Pearson's correlation between two normally distributed variables was determined. For non-normally distributed variables, comparisons between 2 groups were analyzed using the Mann-Whitney U test. Spearman's correlation between two non-normally distributed variables was determined. The Spearman *r* value and 95% confidence interval (CI) are shown. A binary logistic regression model and a linear regression model of log_10_ transformed variables were constructed. P<0.05 was considered significant; 0.05≤p<0.10 was considered a significant trend. Values are presented as the median [95% CI of the median].

## Results

### Study Subject Characteristics

We enrolled 36 consecutive PUUV-infected patients who presented to Tampere University Hospital in prospective fashion. Serial daily blood and urine samples were collected in all study subjects throughout their hospital course, and at convalescent visits 2 weeks and 6 months after hospitalization. 15 patients (42%) developed severe AKI; two patients required hemodialysis support, and all patients recovered from their PUUV infection without serious sequelae.

**Figure 1:**
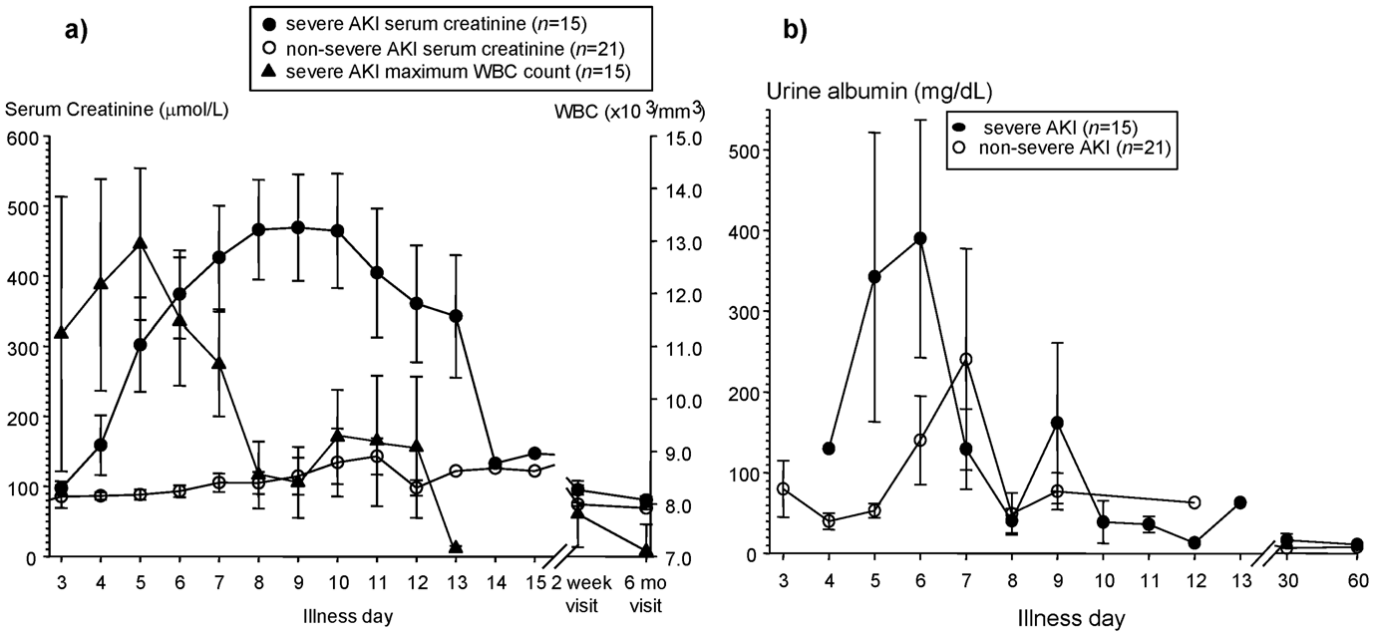
The rise in serum creatinine (Cr) is higher in Puumala virus (PUUV)-infected patients with severe acute kidney injury (AKI) compared to those with non-severe AKI. The maximum white blood cell (WBC) count occurred several days before the peak Cr in PUUV-infected patients with severe AKI (a). PUUV-infected patients with severe AKI have greater proteinuria than those with non-severe AKI (b). Severe AKI is >3-fold rise in serum Cr during acute illness compared to a 6 month baseline. Illness day 1 is the first calendar day of reported fever. Symbols and error bars are mean±S.E.

There were no significant differences in the baseline characteristics and days of fever at clinical presentation between the patients with severe AKI and those with non-severe AKI (Table 1). The peak fold-change in serum Cr was 7.6 [5.4–9.7] among the patients with severe PUUV-induced AKI and 1.7 [1.4–2.0] among the patients with non-severe PUUV-induced AKI. Among the patients with severe PUUV-induced AKI, serum Cr levels typically peaked 8–10 days after illness onset whereas maximum levels of proteinuria were generally seen 5–7 days after illness onset (Figure 1). As expected, patients with severe PUUV-induced AKI had a longer duration of hospitalization, higher systolic and diastolic blood pressures, more severe oliguria, and greater weight change compared to those with non-severe PUUV-induced AKI (Table 1).

**Table 1:** Characteristics of study subjects with PUUV nephropathia epidemica.

	Non-severe AKI^a^ (*n* = 21)	Severe AKI^a^ (*n* = 15)	Univariate p-value
Fold-change in serum creatinine^b^ (sCr) (peak sCr/baseline sCr)	1.7 (1.4, 2.0)	7.6 (5.4, 9.7)	---
Age (y)^b^	45 (39, 50)	46 (40, 53)	1.0
Gender (M∶F)	12∶9	12∶3	0.3
Number of days of illness at time of hospital admission^b^	4 (4, 5)	5 (4, 5)	0.4
Duration of hospitalization (days)	5 (4, 6)	8 (6, 9)	**0.001**
Tobacco smokers (%)	38%	60%	0.3
BMI (kg/m^2^)^b^	23.3 (19.4, 27.2)	27.9 (25.8, 30.1)	0.08
Maximum systolic blood pressure^b^ (mm Hg)	134 (129, 140)	151 (140, 162)	**0.02**
Minimum systolic blood pressure^b^ (mm Hg)	111 (104, 117)	122 (112, 131)	**0.04**
Maximum diastolic blood pressure^b^ (mm Hg)	86 (81, 91)	93 (88, 99)	**0.04**
Minimum diastolic blood pressure^b^ (mm Hg)	68 (62, 73)	69 (64, 74)	0.7
Minimum urine output^b^ (ml/24 h)	1,521 (1,084, 1,959)	955 (577, 1,332)	**0.049**
Δweight^b^(kg)	1.6 (1.1, 2.1)	4.2 (2.5, 5.8)	**<0.001**

a AKI = acute kidney injury; severe AKI = >3-fold rise in serum creatinine during acute illness compared to a 6 month baseline value.

b Values are median (95% confidence interval of median).

### Leukocytosis is a risk factor for severe PUUV-induced AKI

PUUV nephropathia epidemica is often characterized by a moderate leukocytosis, thrombocytopenia, elevated C-reactive protein (CRP) levels, and a mild transaminitis [11], [12]. We found that maximum white blood cell (WBC) counts were significantly higher in PUUV-infected patients who developed severe AKI compared to those with non-severe AKI (Table 2), and the highest WBC levels were generally seen 3–6 days after illness onset (Figure 1a). The maximum leukocyte count was positively correlated with the peak fold-change in serum Cr, regardless of AKI severity classification (Spearman *r* = 0.54 [0.26–0.74], p = 0.001). By contrast, there were no significant associations between PUUV-induced AKI severity and platelet counts, CRP levels, or alanine aminotransferase (ALT) levels (Table 2).

**Table 2:** Clinical laboratory indicators in study subjects with PUUV nephropathia epidemica.

	Non-severe AKI^a^ (*n* = 21)	Severe AKI^a^ (*n* = 15)	Univariate p-value
Maximum leukocyte count^b^ (×10^6^/mm^3^)	9.2 (8.1, 10.3)	13.4 (10.9, 15.9)	**0.001**
Minimum platelet count^b^ (×10^3^/mm^3^)	58 (47, 69)	59 (42, 75)	0.9
Maximum C-reactive protein level^b^ (mg/L)	103 (80, 125)	91 (71, 111)	0.5
Maximum alanine aminotransferase (ALT) level^b^ (U/ml)	64 (40, 88)	58 (44, 71)	0.5

a AKI = acute kidney injury; severe AKI = >3-fold rise in serum creatinine during acute illness compared to a 6 month baseline value.

b Values are median (95% confidence interval of median).

### Urine IL-6 and IL-8 levels are positively correlated with AKI severity in PUUV nephropathia epidemica

**Figure 2:**
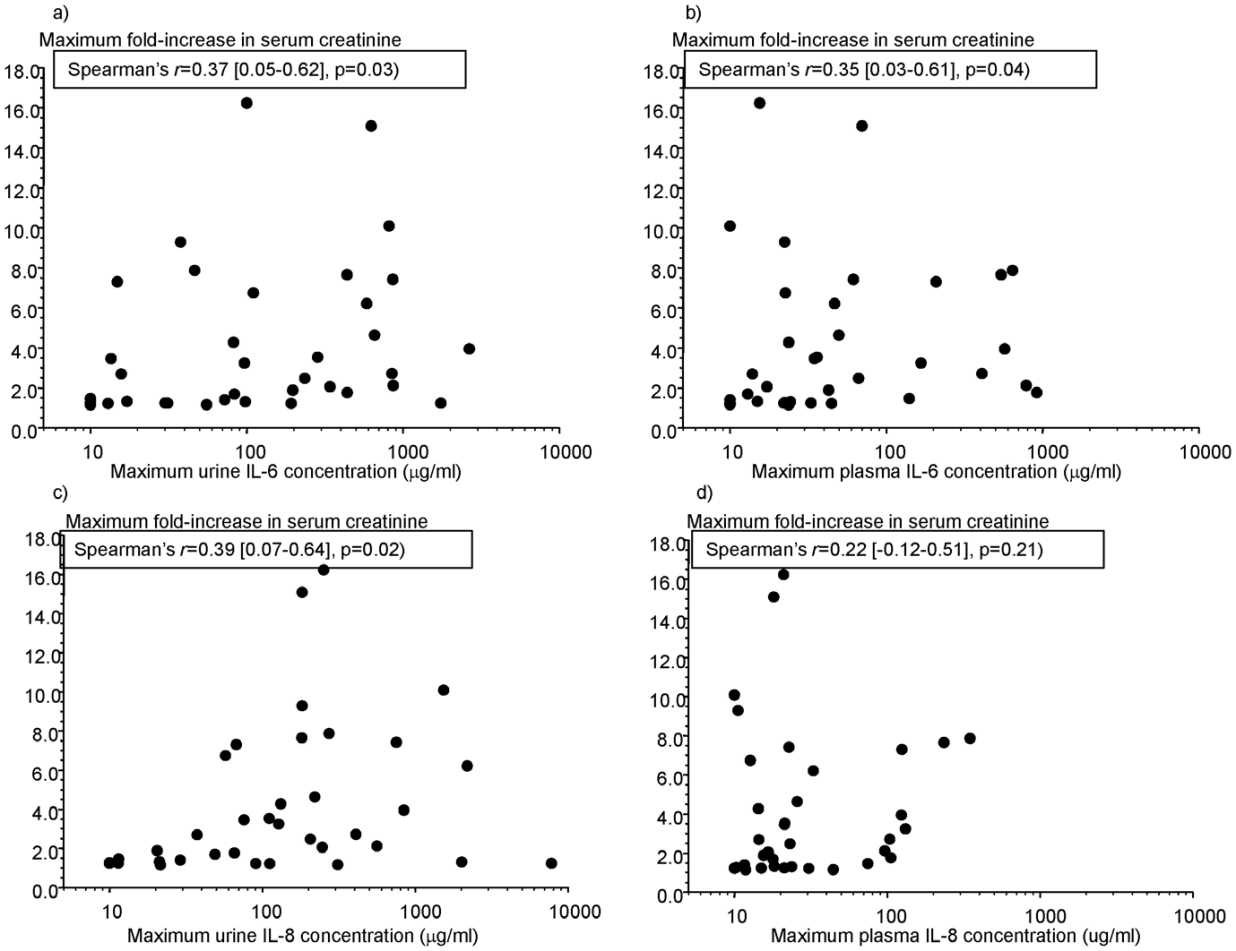
Maximum urine IL-6, plasma IL-6, and urine IL-8 concentrations are positively correlated with the degree of PUUV-induced AKI. a) maximum urine IL-6 levels b) maximum plasma IL-6 levels c) maximum urine IL-8 levels d) maximum plasma IL-8 levels.

In an earlier study, high plasma interleukin (IL)-6 levels were found to be associated with clinically severe PUUV NE [6]. We measured the serial daily urine and plasma concentrations of 8 cytokines and chemokines over the course of acute PUUV infection (IL-1α, IL-1β, IL-2, IL-6, IL-8, IL-15, CXC Ligand 10 (CXCL10), and monocyte chemoattractant protein-1 (MCP-1)). The maximum levels of IL-6 in urine and plasma during acute illness were positively correlated with PUUV-induced AKI severity. Maximum urine IL-8 concentrations, but not plasma IL-8 concentrations, were also positively associated with PUUV-induced AKI (Figures 2a–d). We did not find any significant associations between the degree of PUUV-induced AKI and urine or plasma levels of IL-1α, IL-1β, IL-2, IL-15, CXCL10, or MCP-1. Urine cytokine associations did not differ whether the levels were expressed as a concentration or fractional excretion.

### Urinary sediment GATA-3 mRNA levels are positively correlated with AKI severity in PUUV nephropathia epidemica

**Figure 3:**
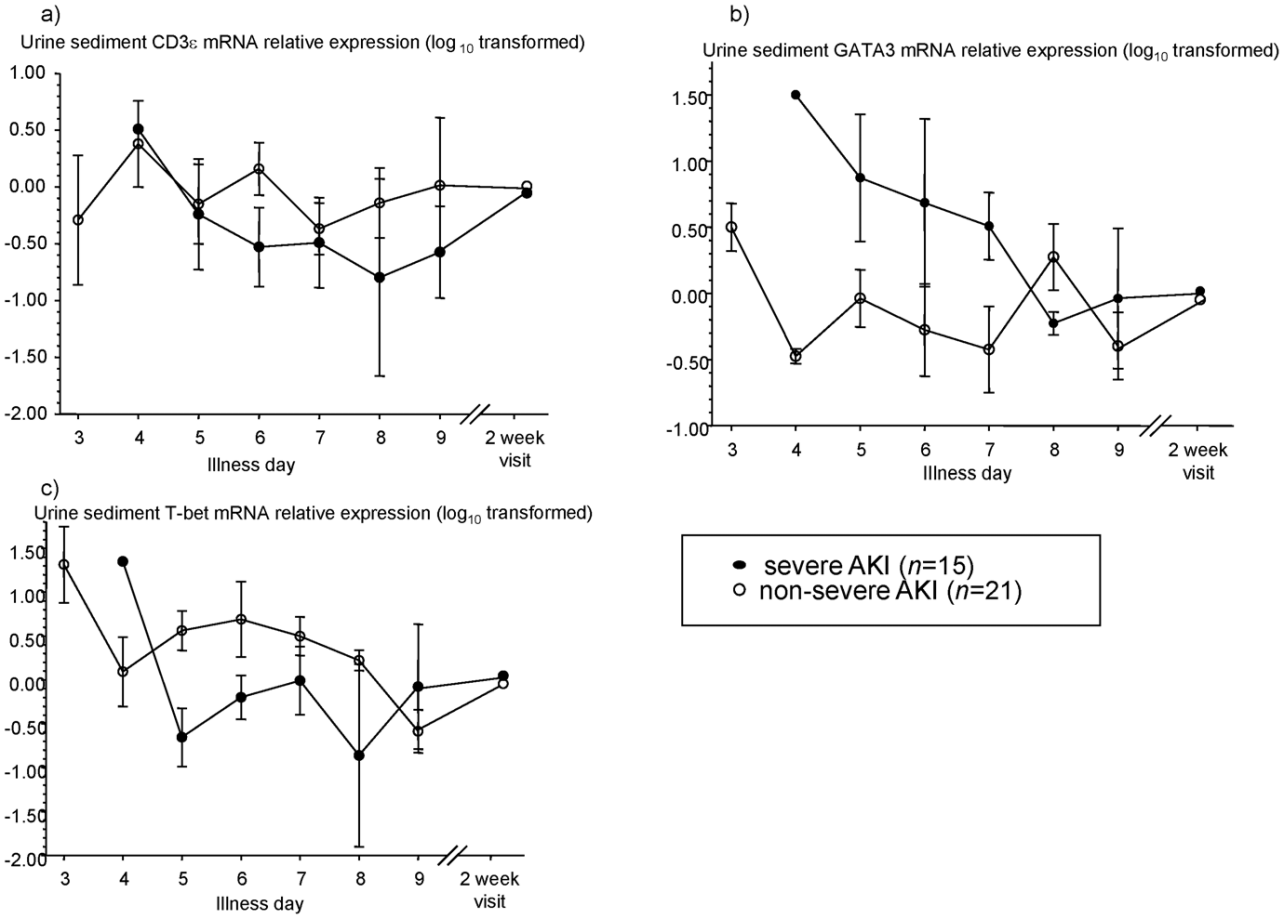
Urinary sediment GATA-3 mRNA levels are elevated and T-bet mRNA levels are decreased during acute illness in PUUV-induced severe AKI. Severe AKI is >3-fold rise in serum Cr during acute illness compared to a 6 month baseline. Illness day 1 is the first calendar day of reported fever. a) Urinary sediment CD3ε mRNA relative expression compared to 2 week baseline value b) Urinary sediment GATA-3 mRNA relative expression compared to 2 week baseline value c) Urinary sediment T-bet mRNA relative expression compared to 2 week baseline value. Symbols and error bars are mean±S.E.

In an attempt to characterize the local and renal-specific immune responses in acute PUUV infections, we measured mRNA expression levels of a T-cell associated gene (CD3ε), a type 1 cytokine transcription factor (T-bet), and a type 2 cytokine transcription factor (GATA-3) in serial urinary sediment samples. The levels of CD3ε, T-bet, and GATA-3 mRNA in urinary cells during acute illness were expressed relative to 2 week convalescent values in each patient. Urinary sediment CD3ε mRNA expression levels were essentially unchanged throughout acute illness and were not significantly different between patients with severe and non-severe AKI (Figure 3a). In patients with severe AKI, urinary sediment GATA-3 mRNA expression was increased, and T-bet mRNA expression was decreased, during acute illness compared to 2 week convalescent values (Figures 3b&c). Maximum urinary sediment GATA-3 mRNA levels were significantly higher in PUUV infected patients who developed severe AKI compared to those with non-severe AKI. The maximum urinary sediment GATA-3 mRNA level was positively correlated with the peak fold-change in serum Cr, regardless of AKI severity classification (Spearman's *r* = 0.53 [0.21–0.75], p = 0.003).

### Elevated peripheral WBC counts and urinary sediment GATA-3 mRNA levels are independent risk factors for severe AKI in PUUV nephropathia epidemica

On univariate analysis, maximum peripheral WBC, urine IL-8, and urinary cell GATA3 mRNA levels were associated with severe AKI in PUUV infections. In a multivariate logistic regression model, maximum urinary cell GATA-3 mRNA levels were an independent risk factor for severe AKI in PUUV nephropathia epidemica. The maximum WBC trended significantly with PUUV-induced severe AKI (Table 3). Among patients with severe AKI, the maximum urinary cell GATA-3 mRNA level occurred 1 day [0–4 days] (median [95% CI]) before the maximum serum Cr level, whereas the maximum WBC count occurred 2 days [0–4 days] (median [95% CI]) before the maximum serum Cr. In a linear regression model, the log_10_ maximum WBC and log_10_ maximum urinary cell GATA-3 mRNA were significantly correlated with the log_10_ transformed peak Cr ratio (Table 3).

**Table 3:** Multivariate statistical models for acute kidney injury in study subjects with PUUV nephropathia epidemica.

Multivariate logistic regression model			
Variable	Odds Ratio for severe AKI^a^	95% CI^b^	Multivariate p-value
Maximum urinary cell GATA-3 mRNA level^c^	4.7	[1.04–21.0]	**0.045**
Maximum WBC count^c^^, ^ d	6.2	[0.9–42.5]	0.065
Maximum urine IL-8 concentration^c^	2.2	[0.6–8.8]	0.27

Linear Regression Model			
Variable	Linear regression coefficient^e^	95% CI^f^	Multivariate p-value
Maximum WBC count^c^^, ^ d	1.1	[0.13–2.1]	**0.03**
Maximum urinary cell GATA3 mRNA level^c^	0.14	[0.02–0.26]	**0.02**
Maximum urine IL-8 concentration^c^	−0.003	[−0.27–0.27]	0.98
Maximum urine IL-6 concentration^c^	0.07	[−0.19–0.32]	0.59
Maximum plasma IL-6 concentration^c^	0.036	[−0.15–0.22]	0.70

a Odds ratio (OR) for developing severe PUUV-induced acute kidney injury (AKI) compared to non-severe AKI. Severe AKI = >3-fold rise in serum creatinine during acute illness compared to a 6 month baseline value.

b 95% confidence interval (CI) for the Odds Ratio.

c log_10_ transformed variable.

d White blood cell (WBC).

e Linear regression of log_10_ transformed peak creatinine ratio vs. log_10_ transformed variables.

f 95% confidence interval (CI) for the linear regression coefficient.

## Discussion

In this study, 36 patients presenting with PUUV infection were prospectively followed throughout acute illness, and 2 weeks and 6 months after hospital discharge. During the course of their acute PUUV infection, the patients developed either non-severe or severe AKI as defined by serum Cr levels (severe AKI = >3 fold rise in serum Cr during acute illness compared to 6 month baseline value [10]). As expected, PUUV infected patients who developed severe AKI had a longer duration of hospitalization, greater changes in blood pressure, urine output, and weight than those with non-severe AKI.

To assess potential risk factors for severe AKI in PUUV NE, we first examined common clinical laboratory indicators. Nearly all PUUV NE patients had elevated WBC counts, a decrease in platelet counts, and increases in CRP and ALT levels during acute illness. Only the maximum WBC count correlated with the severity of AKI on univariate and multivariate analyses. Leukocytosis likely reflects the renal inflammatory process during PUUV infection. The maximum WBC count occurred 2 days [0–4 days] (median [95% CI]) before the maximum serum Cr during acute illness. Similar to previous reports, we found that CRP levels did not correlate with the severity of PUUV induced AKI [12].

Serial levels of eight cytokines and chemokines (IL-1α, IL-1β, IL-2, IL-6, IL-8, IL-15, CXCL10 and MCP-1) were measured daily in both urine and plasma during acute PUUV NE. Only maximum urine IL-6 and IL-8 levels, and maximum plasma IL-6 levels, positively correlated with AKI severity in PUUV infected patients. Serum and urine levels of IL-6 have been previously reported to correspond with PUUV-induced AKI severity [6], [7], [13]. The univariate correlation between maximum urine IL-8 concentrations and PUUV-induced AKI severity is a novel finding and suggests a local inflammatory reaction in PUUV infected kidneys. Kidney biopsies of patients with PUUV NE revealed histological evidence of local inflammation and increased expression of several cytokines in the peritubular area of the distal nephron [11]. However, none of the currently tested cytokines or chemokines were independent risk factors for PUUV-induced AKI severity on multivariate analysis.

In an attempt to characterize local and renal-specific immune responses in acute PUUV infections, we next measured mRNA expression levels of a T-cell receptor associated gene (CD3ε), a type 1 cytokine transcription factor (T-bet), and a type 2 cytokine transcription factor (GATA-3) in serial urinary sediment samples. Only maximum GATA-3 mRNA levels in urinary sediment correlated with the severity of AKI on univariate and multivariate analyses. The maximum urinary sediment GATA-3 mRNA level occurred 1 day [0–4 days] (median [95% CI]) before the maximum serum Cr during acute illness. GATA-3 is a necessary transcription factor for the generation of type 2 T-cells [9], [14], and severe AKI in PUUV infection might be due to excessive type 2 T-cell responses compared to type 1 T-cells in the kidney. Excessive type 1 T-cell kidney infiltration and T-bet urinary sediment levels have been implicated in lupus nephritis [15], proliferative and crescentic glomerulonephritis [16], and acute kidney transplant rejection [17]. Elevated GATA-3 mRNA levels in circulating mononuclear cells have been reported in patients with minimal change nephrotic syndrome [18]. In addition to type 2 T-cells, the GATA-3 transcription factor is expressed in human kidney embryogenesis [19], and by distal renal tubular and collecting duct cells in the adult kidney [20]. The T-cell receptor associated gene (CD3ε) urinary sediment mRNA level was essentially unchanged throughout acute illness, and there was no difference between patients with severe and non-severe AKI due to PUUV infections. Therefore, we postulate that the sources of elevated GATA-3 mRNA levels in the urinary sediment of patients with severe PUUV-induced AKI were distal renal tubular or collecting duct cells. Another possibility is that the cellular sources of GATA-3 mRNA in the urinary sediment changed over time. In our study protocol, we were unable to definitively identify the cellular sources of GATA-3 mRNA expression in urinary sediment. We suspect that elevated GATA-3 mRNA urinary sediment levels in patients with severe AKI reflect kidney injury in distal tubular or collecting duct cells in PUUV-infected kidneys. Additional studies and patients are required to identify whether the urinary sediment GATA-3 mRNA levels in PUUV infection reflect type 2 T-cell responses or distal tubular kidney injury.
